# Clinical significance of sunitinib‐associated macrocytosis in metastatic renal cell carcinoma

**DOI:** 10.1002/cam4.919

**Published:** 2016-10-19

**Authors:** Maria T. Bourlon, Dexiang Gao, Sara Trigero, Julia E. Clemons, Kathryn Breaker, Elaine T. Lam, Thomas W. Flaig

**Affiliations:** ^1^ Division of Medical OncologyInstituto Nacional de Ciencias Médicas y Nutrición Salvador ZubiránMexico CityMexico; ^2^ Department of Biostatistics and InformaticsUniversity of ColoradoDenverColorado; ^3^ School of MedicineUniversity of Colorado DenverAuroraColorado; ^4^ Division of Medical OncologyDepartment of MedicineSchool of MedicineUniversity of ColoradoAuroraColorado; ^5^ University of Colorado Cancer CenterAuroraColorado

**Keywords:** Clinical biomarker, macrocytosis, mean corpuscular volume, progression‐free survival, renal cell carcinoma, sorafenib, sunitinib, tyrosine kinase inhibitors

## Abstract

Increases in the mean corpuscular volume (MCV) have been observed in patients with metastatic renal cell carcinoma (mRCC) on tyrosine kinase inhibitor (TKI) treatment; however, its association with progression‐free‐survival (PFS) is unknown. We aimed to characterize TKI‐associated macrocytosis in mRCC and its relationship with PFS. Retrospective review of data on macrocytosis and thyroid dysfunction on mRCC patients treated with sunitinib and/or sorafenib. These results are evaluated in the context of our previous report on the association of hypothyroidism in this setting. We assessed PFS as clinically defined by the treating physician. Seventy‐four patients, 29 of whom received both drugs, were included. A treatment period was defined as time from initiation to discontinuation of either sunitinib or sorafenib; 103 treatment periods [sorafenib (47), sunitinib (56)] were analyzed. Macrocytosis was found in 55 and 8% of sunitinib‐ and sorafenib‐treated patients, respectively, *P* < 0.001. The median time to developing macrocytosis was 3 months (m, range 1–7). Median PFS in sunitinib‐treated patients was 11 m (95% CI: 6–19). Median PFS was higher among those with macrocytosis compared to normocytosis (21 m [95% CI: 11–25] vs. 4 m [95% CI: 3–8] *P* = 0.0001). Macrocytosis and hypothyroidism were two significant predictors of PFS. The greatest difference in PFS among all patients was observed in patients with both macrocytosis and hypothyroidism (25 m), compared to the normocytic and euthyroid patients (5 m) (*P* < 0.0001). Sunitinib‐related macrocytosis was associated with prolonged PFS, and concurrent development of hypothyroidism and macrocytosis further prolonged PFS. Increased MCV may have a role as a predictive biomarker for sunitinib. Prospective studies accounting for other known prognostic factors are needed to confirm this finding.

## Introduction

Renal cell carcinoma (RCC) is the most common type of kidney cancer in adults 1. Up to 30% of patients have metastatic disease at the time of initial diagnosis 2. Vascular‐targeted therapies are standard medical treatment for mRCC 3. Sorafenib and sunitinib were the first tyrosine kinase inhibitors (TKI) to be approved for advanced kidney cancer 4, 5. Sunitinib is commonly utilized as first‐line treatment option 6. Sorafenib has recently shown to increase progression‐free survival (PFS) compared to placebo, and overall survival compared to mTOR inhibitors as second‐line treatment 7, 8.

Sunitinib primarily suppresses vascular endothelial growth factor receptor (VEGFR) types 1–3, as well as, platelet‐derived growth factor receptors alpha and beta (PDGFR‐*α* and PDGFR‐*β*), the stem cell factor c‐KIT, FMS‐like tyrosine kinase 3 (FLT3), and glial cell line‐derived neurotrophic factor receptor (RE arranged during transfection [RET]) 9. Sorafenib predominantly inhibits BRAF, VEGF, and RET; it is a weak c‐KIT inhibitor. The main side effects of both drugs include rash, hand and foot syndrome, mucosal inflammation, nausea, vomiting, diarrhea, hypertension, and fatigue 10, 11. In addition, hypothyroidism occurs in 30–85% for patients treated with sunitinib, and 20–36% of those treated with sorafenib 12, 13.

Elevation of MCV values has been observed with sunitinib, but is uncommon with sorafenib therapy 14. In addition, patients who have hypothyroidism tend to have a greater MCV increase than those who remain euthyroid 15. Nearly half of the mRCC patients treated with sunitinib develop macrocytosis after a median of three cycles, with up to 82% of those who remain normocytic trending toward increased MCV 16. MCV generally returns to normal values at 2–3 m after drug withdrawal without other intervention, suggesting a temporary and reversible nature of this phenomenon. Interestingly, sunitinib‐associated macrocytosis has been reported in other tumor types as well, including breast cancer 14. The predictive significance or impact on survival of sunitinib‐associated macrocytosis remains unknown.

Predictive biomarkers have been well established for treatment in other cancer types. For example, trastuzumab and crizotinib have both demonstrated survival advantage in human epidermal growth factor receptor 2 (HER2)‐positive breast cancer and anaplastic lymphoma kinase (ALK) positive non‐small‐cell lung cancer, respectively 17, 18. For mRCC, there are currently no molecular predictive markers available to guide treatment decisions 19. Identification of predictive biomarkers may allow individualization of therapy in patients with mRCC. Current prognostic models, such as the MSKCC and the IDMC criteria are based on patient′s baseline clinical status and labs. MSKCC criteria are based on time on time from the diagnosis to systemic treatment, hemoglobin level, serum calcium, LDH value, and performance status. The Heng score for mRCC predicts median survival in patients treated with VEGF‐targeted therapy 20, 21. MCV increase offers an on‐treatment marker that can be easily assessed by the clinician during TKI treatment.

Clinical side effects have been evaluated as possible predictive biomarkers to response in mRCC 22. Hypertension, hand and foot syndrome, and hypothyroidism have been associated with improved progression‐free survival and overall survival in mRCC patients treated with sunitinib therapy 23, 24, 25, 26, 27, 28. Similarly, sunitinib‐associated neutropenia was predictive of improved PFS regardless of dose adjustment 29. To the best of our knowledge, the correlation between macrocytosis and PFS has not been reported in mRCC patients treated with TKIs.

Previous studies evaluating the individual effects of sunitinib and sorafenib on thyroid function have shown that the development of hypothyroidism with both drugs was associated with prolonged PFS 26, 30, 31, 32, 33, 34. We also have previously reported on thyroid dysfunction in patients treated with these agents in the same cohort of patients studied here. 27 In this study, our goals were to determine whether the development of macrocytosis was also associated with prolonged PFS and to evaluate both macrocytosis and hypothyroidism with respect to PFS in these patients.

## Materials and Methods

### Patients

A retrospective chart review was performed on all patients with histologically confirmed mRCC treated with sunitinib or sorafenib seen at the University of Colorado Hospital Cancer Center, from January 1, 2005 to January 1, 2011. All patients received either sunitinib or sorafenib as first‐line treatment. Patient with previous systemic therapies were excluded. Patients were excluded if there was insufficient follow‐up information beyond 3 months of TKI initiation. Patients with baseline macrocytosis or microcytosis were excluded.

### Treatment

Patients received standard doses of either sunitinib 50 mg daily (4 weeks on treatment, 2 weeks off) or sorafenib, 400 mg twice daily, continuously. A treatment period was defined as time from initiation to discontinuation of either sunitinib or sorafenib. Ten patients were started at a reduced dose based on the treating physician's clinical assessment. Dose interruptions and adjustments for toxicity or intolerance were performed according to the manufacturer's recommendations. PFS was defined as time from start of treatment until therapy was discontinued due to disease progression as clinically defined by the treating provider. Computed tomography (CT) scans to assess for response were generally performed every 2–3 cycles, although formal Response Evaluation Criteria in Solid Tumors (RECIST) criteria for radiographic progression were not used to define PFS in this retrospective study. Disease progression was based on the treating physician's assessment of radiographic and clinical changes. All treated patients were evaluated and treated by one of two medical oncologists specializing in urologic oncology with the same standard approach.

### Data

Patient charts were examined for demographic data, treatment details, time from start of treatment to disease progression, and history of anemia. Laboratory data including MCV, hemoglobin, and RDW were collected along with TSH, vitamin B12, methylmalonic acid, and folate when available. Macrocytosis was defined as MCV >100 fL. Time from the start of treatment to the development of macrocytosis was also recorded. Thyroid dysfunction data were obtained from our previous reported data 27. Patients were excluded if there was missing or insufficient follow‐up information, including a lack of a baseline TSH value or TSH values during treatment. Hypothyroidism was defined as any TSH increase above the upper limit of normal (5.0 mIU/L). Thyroid replacement was started at the treating physician's discretion, generally if the TSH level was elevated and/or if the patient had clinically significant symptoms thought to be due to hypothyroidism.

### Statistical methods

Patient demographics were described and the differences between sunitinib‐treated and sorafenib‐treated patients were analyzed using a chi‐square test or Fisher's exact test for categorical variables, and two‐group *t*‐test for continuous variables. Kaplan–Meier estimate approach was used to estimate the median PFS for each group evaluated (sunitinib vs. sorafenib; macrocytic vs. normocytic; macrocytic with vs. without hypothyroidism). Log‐rank test was used to compare the corresponding PFS functions among the groups. Cox proportional hazard regression model was used to estimate the hazard ratio (HR) for the development of macrocytosis between the two drugs, as well as the HR of peak MCV on PFS for sunitinib‐treated patients. The effect of macrocytosis on PFS was assessed using time‐dependent Cox model to adjust for the time a patient on sunitinib and when macrocytosis was developed. SAS 9.3 (SAS Inc. Cary, NC) was used for all the analyses. A *P*‐value ≤0.05 was considered statistically significant throughout this study.

## Results

Seventy‐four patients met inclusion criteria with sufficient data for analysis. Twenty‐nine patients received sequential therapy with both sunitinib and sorafenib during the study period. Forty‐seven patients treated with sorafenib and 56 patients who received sunitinib were included. A total of 103 treatment periods were evaluated, 69 of which had complete data on MCV and thyroid function for analysis. The washout periods were adequate and there was no clear evidence of a cross‐over effect in the studied population. Table 1 shows baseline characteristics of the study population.

**Table 1:** Patient characteristics

	Sunitinib (*n* = 56)	Sorafenib (*n* = 47)	*P*‐value
Mean age (SD)	60 (8.2)	59 (9.6)	0.73
Men, no. %	39 (70%)	37 (79%)	0.12
Prior sunitinib therapy	–	10	
Prior sorafenib therapy	19	–	
Macrocytosis at baseline	0	0	

Thirty‐nine percent of patients required at least one dose reduction during their treatment. Over the treatment course, 31 sunitinib‐treated patients developed macrocytosis (55.4%). In contrast, only four out of the 47 patients (8%) who received sorafenib developed macrocytosis. For the sunitinib group, vitamin B12 and folate (by either folate level or RBC folate level) data were available for only 13 and 10 patients, respectively. One of these patients had a low vitamin B12 level, but this patient did not have an elevated MCV. The median time to develop macrocytosis was 3 m (range from 1 to 7 m). The time to development of macrocytosis did not affect the degree of macrocytosis observed (*P* = 0.47).

The effect on hemoglobin differed among the sunitinib and sorafenib groups, with a mean decrease of 1.3 g/dL in the sunitinib group and an increase of 0.06 g/dL in sorafenib‐treated patients from start to end of treatment (*P* = 0.01). Although a decrease in the average hemoglobin was seen at time of development of macrocytosis with respect to baseline, this change was not significant (*P* = 0.70) (Table 2). In patients who developed macrocytosis and remained on therapy, the macrocytosis had resolved later in the course of their ongoing tyrosine kinase inhibitor therapy in 37% and 75% of patients treated with sunitinib and sorafenib, respectively. Macrocytosis resolved in all patients after stopping the drug, with a median time to resolution of 0 m (range 0–3 m) and 1 m (range 0–5 m) for the sorafenib and sunitinib groups, respectively. (Table**** 3).

**Table 2:** Hematologic changes observed in patients on sunitinib and sorafenib

	Sunitinib (*n* = 56)	Sorafenib (*n* = 47)	*P*‐value
Pts who developed Macrocytosis, *n* (percentage %)	31 (55.36%)	4 (8.51%)	<0.0001
Median time to develop macrocytosis, months (range)	3.00 (1–7)	3.50 (1–8)	<0.001
Hgb at beginning of treatment, g/dL (range)	13.41 (9.2–16.2)	13.06 (9.6–18.0)	0.33
Hgb at peak MCV, g/dL (range)	12.32 (8.7–16.1)	11.93 (8.1–14.4)	0.70
Hgb at end of treatment, g/dL (range)	12.16 (8.8–15.8)	13.12 (8.0–17.4)	0.02

Hemoglobin (Hgb), reference lab value for Hgb 14.3–18.1 g/dL. Mean corpuscular volume (MCV).

*A *t*‐test was used to compare the two groups for all variables. Except, the Median time to develop macrocytosis, which was compared using log‐rank test between the two groups.

**Table 3:** Resolution of macrocytosis in patients with follow‐up

	Sunitinib (*n* = 27)^a^	Sorafenib (*n* = 4)
Macrocytosis resolved while on therapy with tyrosine kinase inhibitor	37%	75%
Macrocytosis resolved during follow‐up	100%	100%
Median time to resolution after stopping therapy	1 m	0 m

a Missing follow‐up information for 4/31 patient patients that explains *n* = 27.

Of the 56 patients treated with sunitinib, 42 had TSH data available. Nineteen patients developed hypothyroidism (45.2%) (Table 4) 27. Thirteen patients (31%) developed both macrocytosis and hypothyroidism and the development of macrocytosis was correlated with hypothyroidism (*P* = 0.02). Patients who developed hypothyroidism tended to have increased mean MCV compared to those who did not develop hypothyroidism (103.8 fl vs. 99.57 fl). The relative risk for developing hypothyroidism for patients without macrocytosis is 0.75 of that for patients with macrocytosis (95% CI, 0.36–1.57).

**Table 4:** Thyroid and hematologic status in sunitinib‐treated patients (available data *n* = 42)

	Developed hypothyroidism (*n* = 19)	Remained euthyroid (*n* = 23)
Developed macrocytosis *n* = 26	13 (31%)	13 (31%)
Remained normocytic *n* = 16	6 (14%)	10 (24%)
Average peak MCV	103.8	99.67

**Figure 1:**
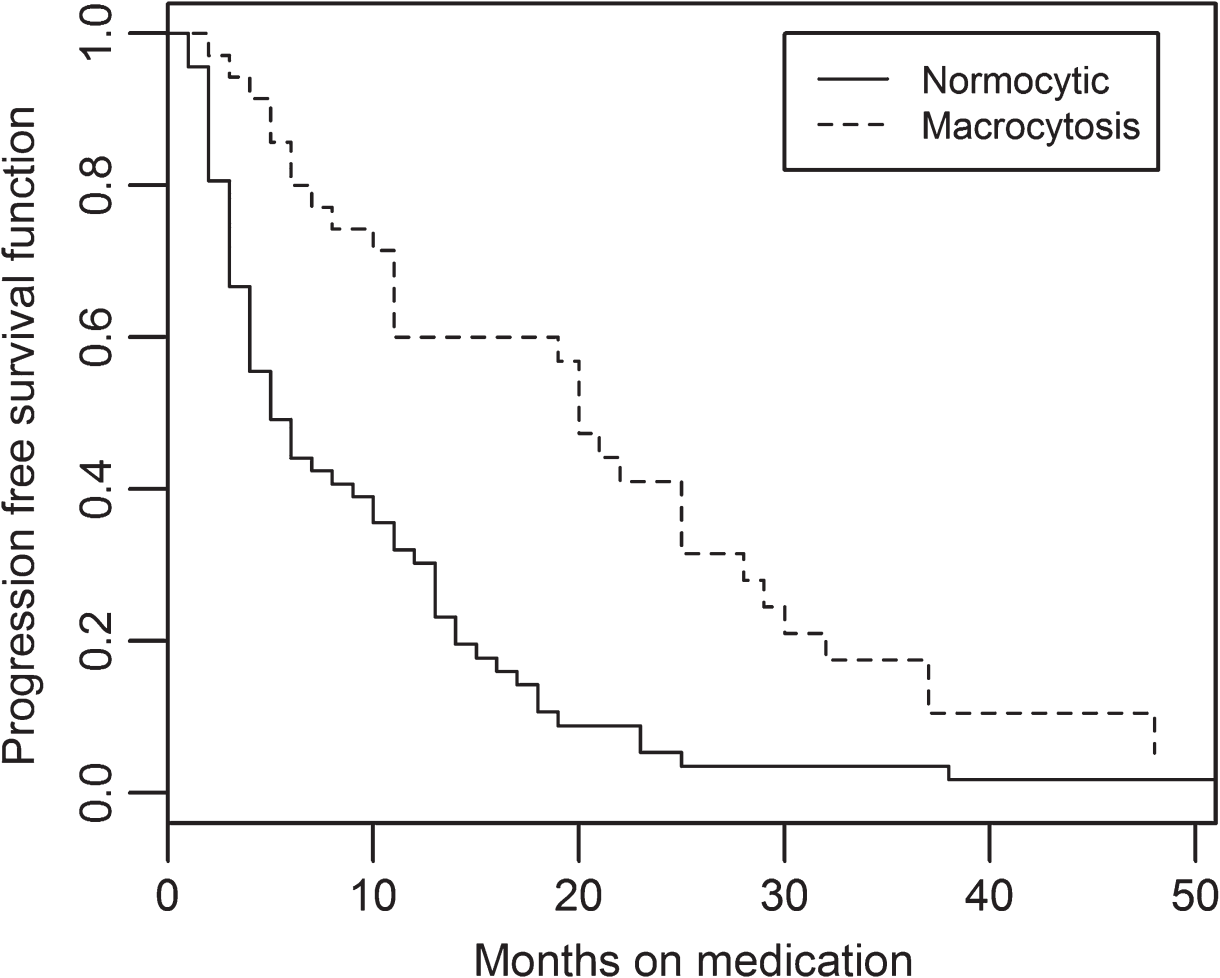
Kaplan–Meier curve of progression‐free survival (PFS) for sunitinib‐treated patients (*n* = 56) based on the development of macrocytosis.

Among all patients treated with sunitinib, the median PFS was 11.6 m (95% CI: 6–19)**.** Median PFS was 21 m (95% CI: 11–25) for macrocytic patients compared to 4 m (95% CI: 3–8) in normocytic patients (*P* = 0.0001) (Fig. 1). The risk of disease progression for patients with macrocytosis is 40% (95% CI,16%, 99%) of that for patients with normocytosis at any fixed point in time (P=0.048). Additionally, the degree of macrocytosis also appeared to confer an effect on PFS with higher peak MCVs significantly associated with longer PFS (*P* < 0.001), with a HR of 0.95, meaning that every unit increase in peak MCV was associated with a 5% decrease in disease progression.

**Figure 2:**
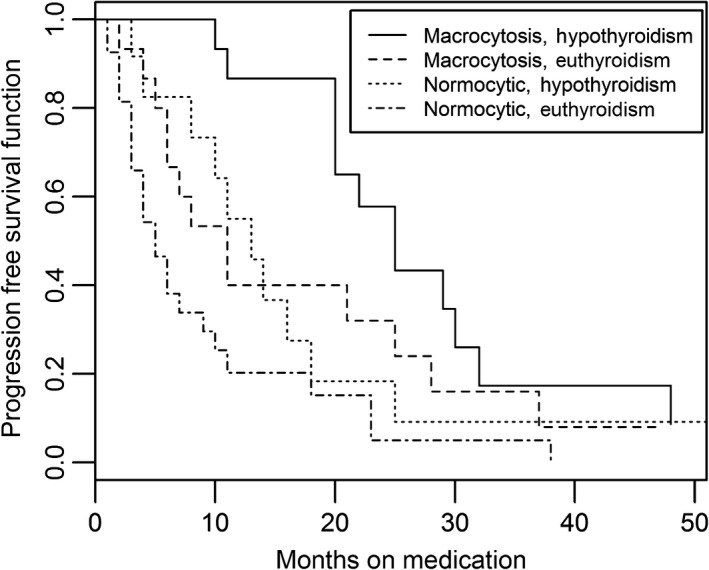
Kaplan–Meier Curve for progression‐free survival (PFS) in patients treated with sunitinib and sorafenib (*P* = 0.005).

**Figure 3:**
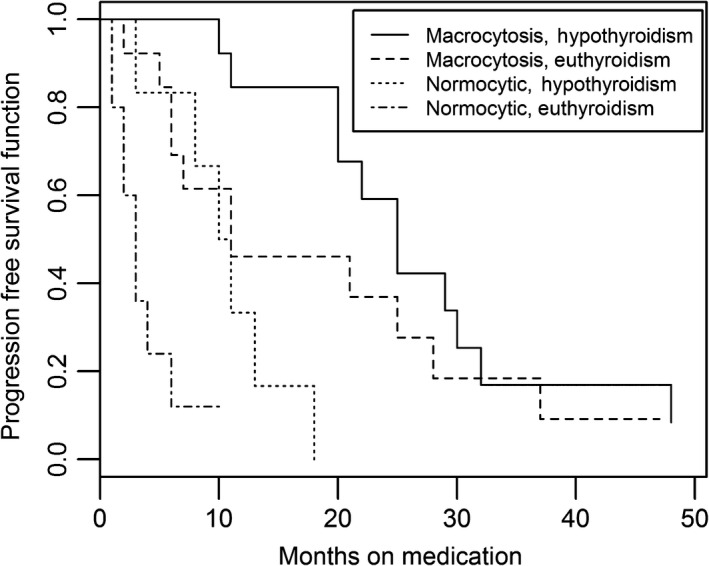
Kaplan–Meier Curve for progression‐free survival (PFS) for sunitinib‐treated patients (*P* < 0.0001).

We also examined the combined influence of MCV and hypothyroidism among patients treated with either sunitinib or sorafenib. We evaluated the interaction of MCV and thyroid function in four designated groups: *Group 1*. Patients with both macrocytosis and hypothyroidism. *Group 2*. Patients with macrocytosis in a euthyroid state. *Group 3*. Patients who were normocytic in a hypothyroid state. *Group 4*. Patients who were normocytic and euthyroid. Among patients treated with sunitinib or sorafenib, PFS was significantly different among the four groups, *P* = 0.0005 by log‐rank test (Table 5 and Fig. 2). In the patients who developed both macrocytosis and hypothyroidism, the median PFS was 25 m (95% CI: 20–32) compared to 5 m in patients who were normocytic and euthyroid (*P* < 0.0001). Macrocytosis and hypothyroidism were two significant predictors of prolonged PFS in our cohort of patients treated with sunitinib or sorafenib. Additionally, the analysis for the sunitinib‐treated group (*n* = 42) also showed the PFS difference among the four groups was statistically significant (*P* < 0.0001 log‐rank test) (Table 6 and Fig. 3). Patients who develop both macrocytosis and hypothyroidism demonstrated the longest PFS (median PFS = 25 m), while patients who did not develop either macrocytosis or hypothyroidism had the shortest PFS (median PFS = 5 m) of any group. This finding was statistically significant (Fig. 2).

**Table 5:** Progression‐free survival (PFS) in the four different groups according to the development of macrocytosis and hypothyroidism for sunitinib and sorafenib

GROUP	Macrocytosis	Hypothyroidism	*N* = 69	PFS (median)	95% CI	*P*‐value
1	Yes	Yes	15	25	20–32	0.0005
2	Yes	No	15	11	5–25	
3	No	Yes	12	13	4–18	
4	No	No	27	5	3–9	

**Table 6:** Progression‐free survival (PFS) in the four different groups according to the development of macrocytosis and hypothyroidism for sunitinib‐treated (*n* = 42 with available data)

GROUP	Macrocytosis	Hypothyroidism	*n* = 42	PFS (median)	95% CI	*P*‐value
1	Yes	Yes	13	25	20–32	<0.0001
2	Yes	No	13	11	6–28	
3	No	Yes	6	10.5	3–18	
4	No	No	10	3	1–6	

## Discussion

In this retrospective study, the development of macrocytosis while on sunitinib therapy was found to be positively correlated with prolonged PFS in mRCC patients. Additionally, those who developed both macrocytosis and hypothyroidism with sunitinib treatment had a better PFS than those that did not. The main limitations of this study are its relatively small size and the retrospective nature with the PFS not strictly defined as in prospective studies, but it was determined in a real‐world clinical setting. Notably, the PFS and rate of macrocytosis in this cohort were similar to data reported elsewhere for patients treated with sunitinib providing assurance of the data quality in this investigation 15, 35, 36. The time to the development of macrocytosis was short (mean of 3 m) suggesting that it could be an early indicator of treatment effect and the time to development of macrocytosis did not affect the degree of macrocytosis observed. This finding will need to be confirmed in larger cohorts and corrected for known baseline prognostic factors in this setting such as the Memorial Sloan Kettering Cancer Center (MSKCC) and International Metastatic Renal Cell Carcinoma Database Consortium (IMDC) criteria. In addition, the assessment of the MCV/survival association was not assessed at prespecified time points; however, we observed that the median time to the develop macrocytosis in the group with sunitinib was 3 months (range 1–7 months) and 3.5 months (range 1–8 months) for those on sorafenib arm.

Our group has previously reported that the median time to developing hypothyroidism was 11 m and 20 m for patients on sunitinib and sorafenib, respectively 27. In contrast, we found that the time to developing macrocytosis occurred at 3 m on average in sunitinib‐treated patients 15. Elevation of the MCV by itself may prove to be an important factor for elucidating a patient's benefit from sunitinib with the advantage of being an inexpensive, and an easily interpretable laboratory value. In our study, longer duration of time to development of macrocytosis was associated with slightly prolonged PFS, so macrocytosis may be subject to the same bias as hypothyroidism, but macrocytosis generally occurred earlier than hypothyroidism in this context and may serve as a more practical marker for predicting overall response. Additionally, the possibility of using both MCV and TSH as markers of therapeutic benefit with sunitinib is certainly interesting and clinically relevant. The independent impact of macrocytosis in relation to other postulated clinical bio‐markers such as hypertension or hand and foot syndrome could not be established in our cohort, given the retrospective nature of the study and available data.

The mechanism of increasing MCV is less well understood than other reported biomarkers of TKI therapy such as hypothyroidism or hypertension. Many mechanisms for the development of hypothyroidism have been postulated, including VEGFR‐ and/or PDGFR‐mediated capillary regression and thyroid atrophy, inhibition of thyroid peroxidase, and development of drug‐induced thyroiditis 37, 38. Hypertension is believed to result from VEGF inhibition and the consequent decrease in nitric oxide synthase activity and decreased nitric oxide production 39. Sunitinib‐associated macrocytosis has been postulated to occur through a direct effect of the drug. It has been observed despite normal vitamin B12 and folate levels 40 and resolves upon treatment discontinuation. Sunitinib has a longer half‐life compared to sorafenib, and this may explain that the median time for resolution of macrocytosis was longer on the sunitinib‐treated group 41. Furthermore, sunitinib does not appear to alter hemoglobin concentrations significantly despite the development of macrocytosis. Inhibition of the c‐KIT‐dependent signaling pathway of erythroid progenitor cells of the bone marrow has been proposed as an explanation. Inhibition of this pathway might affect normal cell differentiation and proliferation in the bone marrow. It has been documented that the c‐KIT inhibitor, imatinib, caused macrocytosis in patients treated for gastrointestinal stromal tumors (GIST). However, at recommended pharmacodynamic dosing for inhibiting the c‐KIT pathway, macrocytosis was more frequent in sunitinib than with imatinib therapy. In addition, in our study, we noted that the incidence of macrocytosis is different among the sunitinib and sorafenib groups, also supporting this development as a sutent‐specific effect. Taken together, this suggests that inhibition of other pathways such as VEGF, FLT, and RET could also be implicated. The particular contribution of other signaling molecules of protein kinases in the development of myeloid and erythroid progenitor cells is being studied 42. Patients did develop a slight decrease in their hemoglobin levels, but the development of macrocytosis was not associated with worsening anemia.

The associate of hypothyroidism on PFS seems to be greater as compared to macrocytosis. However, even when euthyroid, patients with macrocytosis had better PFS compared to those with normocytosis (Fig. 2). Hypothyroidism did appear to correlate with higher MCVs in our study. Those with hypothyroidism tended to have increased mean MCV compared to those who did not develop hypothyroidism (103.8 fl vs. 99.57 fl). Hypothyroidism has been known to cause macrocytosis typically via an association with pernicious anemia that can occur in up to 10% of patients with chronic autoimmune thyroiditis 43. Macrocytosis has been reported in patients with hypothyroidism and may result from the insufficiency of the thyroid hormones themselves without nutritive deficit or signs of megaloblastic changes 44. Therefore, macrocytosis as a result of hypothyroidism alone and not related to drug effect is unlikely at the rate observed in this study. Both hypothyroidism and macrocytosis were independent predictors of prolonged survival in our cohort.

The importance of this study is that macrocytosis may be another clinical biomarker in mRCC patients treated with sunitinib. This is the first time sunitinib‐associated macrocytosis is demonstrated to have a statistically significant association with PFS, the primary endpoint used in the pivotal phase III study establishing sunitinib as a standard of care in mRCC 5. Our observations will need to be confirmed in larger prospective trials or could be tested retrospectively by examining any of the completed, randomized trials of sunitinib in which the MCV was regularly checked as part of routine patient monitoring. This approach will allow for integration of other known baseline prognostic factors such as the MSKCC and IMDC criteria. Additionally, the independent impact of macrocytosis over other clinical biomarkers such as hand and foot syndrome, and hypertension could be evaluated. It is also warranted to consider additionally evaluation of the development of macrocytosis in other clinical settings in which VEGFR TKI's are utilized to better understand the characteristics of the development of TKI‐associated macrocytosis and the correlation with PFS across clinical settings.

## Conclusion

This study demonstrated a significant difference in the incidence of TKI‐associated macrocytosis during treatment with sunitinib versus sorafenib, with a higher incidence of macrocytosis with sunitinib treatment. The development of macrocytosis during sunitinib treatment was associated with statistically significant prolongation of PFS. Macrocytosis may be a potential biomarker for treatment response for sunitinib, but needs to be proven in larger cohorts and adjust the analysis for other known prognostic factors.

## Conflict of Interests

Dr Bourlon, Dr Trigero, Dr Clemons, and Dr Gao have no conflict of interest to disclose. Dr Lam has received clinical trial support (institutional payment for clinical trial costs) from Argos, Bristol Myers Squibb, Exelixis, Peloton, Roche/Genentech, and TRACON. Dr Flaig declares relationships with Pfizer, SmithKline Beecham, Bristol Myers Squibb, Genentech (Institutional payment for clinical trial costs) and Novartis (Institutional payment for clinical trial costs and research support).
